# Preimplantation Genetic Testing for Monogenic Disease of Spinal Muscular Atrophy by Multiple Displacement Amplification: 11 unaffected livebirths

**DOI:** 10.7150/ijms.32319

**Published:** 2019-09-07

**Authors:** Yu Fu, Xiaoting Shen, Haitao Wu, Dongjia Chen, Canquan Zhou

**Affiliations:** 1 Reproductive Medicine Center, The First Affiliated Hospital, Sun Yat-sen University, Guangzhou, China, 510080; 2 Guangdong Provincial Key Laboratory of Reproductive Medicine, Guangzhou, China, 510080; 3 Reproductive Medicine Center, Jiangmen Cental Hospital, Affiliated Jiangmen Hospital of Sun Yat-sen University

**Keywords:** Multiple displacement amplification, haplotype analysis, Spinal muscular atrophy, preimplantation genetic testing for monogenic disease

## Abstract

**Background**: Preimplantation genetic testing for monogenic disease (PGT-M) has become an effective method for providing couples with the opportunity of a pregnancy with a baby free of spinal muscular atrophy (SMA). Multiple displacement amplification (MDA) overcomes the innate dilemma of very limited genetic material available for PGT-M.

**Objective**: To evaluate the use of MDA, combined with haplotype analysis and mutation amplification, in PGT-M for families with SMA.

**Methods**: MDA was used to amplify the whole genome from single blastomeres or trophectoderm (TE) cells. Exon 7 of the survival motor neuron gene 1 (SMN1) and eleven STRs markers flanking the SMN1 gene were incorporated into singleplex polymerase chain reaction (PCR) assays on MDA products.

**Results**: Sixteen cycles (19 ovum pick-up cycles) of PGT-M were initiated in 12 couples. A total of 141 embryos were tested: 90 embryos were biopsied at the cleavage stage and 51 embryos were biopsied at the blastocyst stage. MDA was successful on 94.44% (85/90) of the single blastomeres and on 92.16% (47/51) of the TE cells. And the PCR efficiency were 98.4% (561/570) and 100% (182/182), respectively. In addition, the average allele drop-out (ADO) rates were 13.3% (60/392) and 9.8% (11/112), respectively. The results for SMN1 exon 7 were all matched with haplotype analysis, which allowed an accurate diagnosis of 93.62% (132/141) embryos. Twelve families had unaffected embryos available for transfer and a total of 38 embryos were transferred in 20 embryo transfer cycles. Eight transfers were successful, resulting in a clinical pregnancy rate of 40% (8/20) and an implantation rate of 28.95% (11/38). Finally, 11 healthy babies were born. Among them, 5 SMA carriers were singleton live births and 3 SMA carriers had twin births.

**Conclusion**: Careful handling during the MDA procedure can improve subsequent PCR efficiency and reduce the ADO rate. We suggest that this protocol is reliable for increasing the accuracy of the PGT-M for SMA.

## 1. Introduction

Spinal muscular atrophy (SMA) is a group of autosomal recessive a systemic disorders characterized by degradation of the anterior horn cells of the spinal cord, with subsequent symmetrical proximal muscle weakness and atrophy. SMA has an estimated incidence of 1 in 10,000 live births and an approximate frequency of 1 in 50 for carriers, making it the second most common neuromuscular disease in children after Duchenne muscular dystrophy 1. The causal gene, the survival motor neuron (SMN) gene, which is located on chromosome 5q13, has two highly homologous copies that are both expressed. SMN1 is the telomeric copy and SMN2 is the centromeric copy; these differ only in five intronic and three exonic nucleotides, two of which occur in exons 7 and 8 2,3. Deletions or mutations in SMN1 induce SMA, while the copy number of SMN2 defines its severity. Approximately 95% of SMA patients carry homologous deletions in the SMN1 exon(s) (7 and 8), and the remaining are almost all compound heterozygotes with a deletion on one SMN1 copy and an intragenic mutation in the other copy 4.

Protocols for PGT-M of SMA, based on PCR and restricted enzymatic digestion of SMN1 exon 7 or both exons 7 and 8, have been reported since 1998 5-8, most of which involved two rounds of PCR. Since then, several method, such as fluorescent allele-specific amplification of SMN1 and a minisequencing method, have also been applied to PGT-M of SMA 12-13.

Single-gene disorders require the development and validation of highly sensitive amplification strategies, often using nested polymerase chain reaction (PCR), whole-genome amplification (WGA), or fluorescent PCR methods. The main disadvantages of nested and fluorescent PCR are the difficulty in choosing primers for multiplex PCR and the inefficiency of microsatellite amplification 9. For this reason, a technique is needed that can amplify single-cell DNA with a sufficiently high fidelity for the diagnosis of any known single-gene disorder by standard PCR techniques. One of the most promising techniques is multiple displacement amplification (MDA), as it generates large amounts of template and gives the most complete coverage and unbiased amplification to date; MDA has been used in the PGT-M of many genetic diseases 14-23.

In a previous study, we developed PGT-M protocols to perform direct and indirect analyses simultaneously on single-cell MDA products using singleplex fluorescent PCR of eleven microsatellite markers flanking the SMN1region and SMN1 exon 7. Unlike previous methods, this strategy allowed both the detection of the homologous deletion of SMN1 exon 7 and the discrimination of carrier embryos from normal embryos. MDA was successfully performed in 97.2%(139/143) single cells (80 single lymphocytes and 63 single blastomeres), and the PCR results for the SMN1 exon 7 with MDA products of single lymphocytes agreed with the results of peripheral blood tests. The PCR efficiency was 97.1% (1369/1329) and the allele drop-off (ADO) rate was 9.3% (106/1144), which was much lower than previously reported rates obtained with MDA and a large number of single cells. After the setup was established for single cells, this protocol was used on 12 couples.

## 2. Materials and Methods

### 2.1 Patients

Twelve couples who are heterozygous deletions of exon 7 of the SMN1 gene at risk of transmitting SMA to their offspring attended our clinics, hoping to achieve healthy pregnancies. All 12 couples had had at least one child die from SMA or at least had a history of termination for an affected pregnancy following chorionic villus sampling (CVS). The basic information, reproductive history and embryo biopsy records of all couples are shown in Table 1.

Molecular genetic investigations showed homozygous deletion of exon 7 of the SMN1 gene in the index patient. The parents all had heterozygous deletions of exon 7 of the SMN1 gene.

The couples were required to sign an informed consent form for the PGT-M cycle. The possible risks and potential outcome of the procedure were extensively discussed, and confirmatory prenatal diagnosis for any ensuing pregnancy was recommended. All procedures involving embryo biopsy, single cell diagnosis, and clinical protocols were performed at our center. This study was approved by the Ethics Committee of our hospital.

### 2.2 Pedigree Analysis

Eleven short tandem repeat (STR) markers (D5S435, D5S629, D5S1413, D5S557, D5S637, D5S610, D5S351, D5S681, 5'-MAP1B, D5S1977, and D5S641) closely linked to the SMN1 gene were used to genotype the reproductive partners and their affected children. Primer sequences are given in Table 2. The forward primers were labeled at 5' with 6-FAM, Hex, or Tamra to visualize the corresponding PCR products on an automatic DNA Sequencer (ABI Prism 3100, Applied Biosystems). Both fully informative and semi-informative markers were chosen for molecular analysis in the clinical PGT-M cycles. The allele sizes were obtained from the affected children with high-risk SMA haplotypes. An STR marker was considered informative for the haplotype if both parents were heterozygous for that marker, semi-informative if only one parent was heterozygous, or uninformative if both parents were homozygous or had shared paternal and maternal alleles.

### 2.3 PCR analysis

Each 25 μL reaction system contained 2.5 μL10× PCR buffer, 3 μL of 1/100 diluted MDA products or genomic DNA, 0.2 μM of each primer set, 200 μM dNTPs, 1.5 mM MgCl_2_, and 1 unit of AmpliTaq DNA polymerase (Perkin Elmer, USA). For allele-specific amplification of SMN1 exon 7, the PCR program was 3 min initial denaturation at 96 ºC, followed by 10 cycles of 96 ºC for 30 s, 58.5 ºC for 45, and 72 ºC for 1 min; and 19 cycles of 95 ºC for 30 s, 58.5 ºC for 45 s, and 72 ºC for 1 min, and 72 ºC for 7 min. For markers D5S435, D5S610, D5S557, and D5S681, the program was 95 ºC for 5 min, followed by 36 cycles of 95 ºC for 30 s, 55 ºC for 1 min, and 72 ºC for 1 min, and a final cycle at 72 ºC for 3 min. For markers D5S1413, D5S637, D5S351, 5'-MAP1B, D5S1977, D5S641, and D5S629, the program was 5 min initial denaturation at 95 ºC, followed by 36 cycles of 95 ºC for 30 s, 58 ºC for 1 min, and 72 ºC for 1 min, and final extension at 72 ºC for 7 min. The PCR products were analyzed with an ABI 3100-Avant genetic analyzer.

### 2.4 Ovarian stimulation and ICSI Procedure

Ovarian stimulation, oocyte retrieval, intracytoplasmic sperm injection (ICSI), and embryo biopsy were performed as previously described 25.

### 2.5 Embryo Biopsy

Embryo biopsy for 10 PGT-M cycles ware performed on Day 3 (cleavage stage), and one blastomere was biopsied from each embryo 12. The embryos were cultured further until the blastocyst stage, and the normal blastocysts were vitrified using a Kitazato vitrification kit (Kitazato Biopharma Co. Ltd., Shizuoka, Japan).

Embryo biopsy for the remaining 6 PGT-M cycles was performed on Day 5 or Day 6 (blastocyst stage). A hole (18-20 mm) was created by laser drilling in the zona pellucida of all embryos that showed no sign of hatching on the morning of Day 5. Blastocysts with trophectoderm (TE) cells herniating from the zona pellucida were chosen for biopsy on Day 5 or Day 6. About 5-10 TE cells were aspirated with a biopsy pipette (internal diameter: 30 μm) and dissected with an OctaxShot™ laser system. The blastocysts were vitrified after biopsy 31.

### 2.6 Cell lysates and MDA protocol

Cell lysis and MDA were performed using the reagents and protocol provided by the manufacturer of the Repli-g Midi kit (Qiagen, Germany). Samples were mixed with an additional 3.5 μL of freshly prepared lysis buffer and incubated for 10 min at 65 ºC, followed by addition of 3.5 μL of stop buffer. The obtained solution (10 μL) was used directly for whole genome amplification (WGA) by adding 40 μL of reaction master mix provided in the kit, followed by incubation at 30 ºC for 8 h and subsequent heat inactivation at 65 ºC for 3 min. The amplified DNA was either subjected to PCR immediately or stored at -20 ºC 32.

### 2.7 Embryo transfer

Embryos diagnosed as unaffected were selected for transfer. For cleavage-stage biopsy embryos, embryo transfer was performed on the morning of Day 5, and the remaining normal phenotype blastocysts were frozen. In two cycles, due to the risk of ovarian hyperstimulation syndrome syndrome (OHSS), all embryos diagnosed as unaffected were completely frozen, and embryo transfer was performed in the next frozen-thawed embryos transfer (FET) cycles. All embryos diagnosed as unaffected in the cycles of blastocyst biopsy were transplanted in the next FETcycle. Thawing of unaffected euploid embryos was conducted and the blastocysts were transferred in a FET cycles using standard protocol. Pregnancy outcomes of all couples are shown in Table 3.

## 3. Results

### 3.1 Pedigree analysis

**Figure 1:**
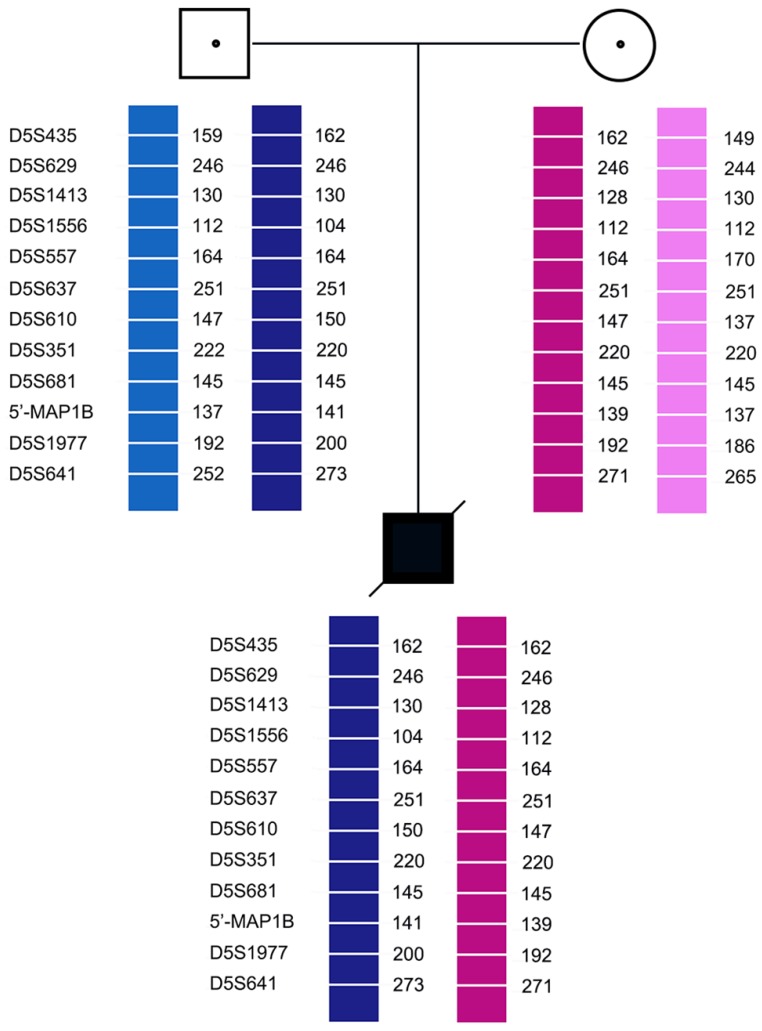
Family analysis.

**Figure 2:**
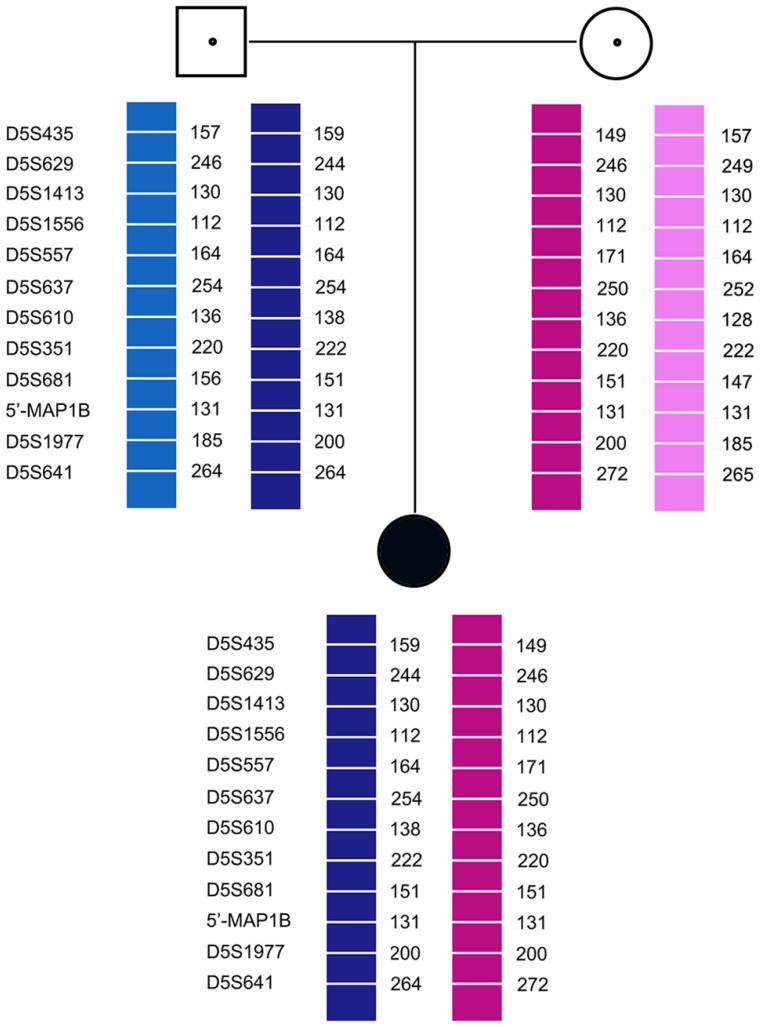
Family analysis.

**Figure 3:**
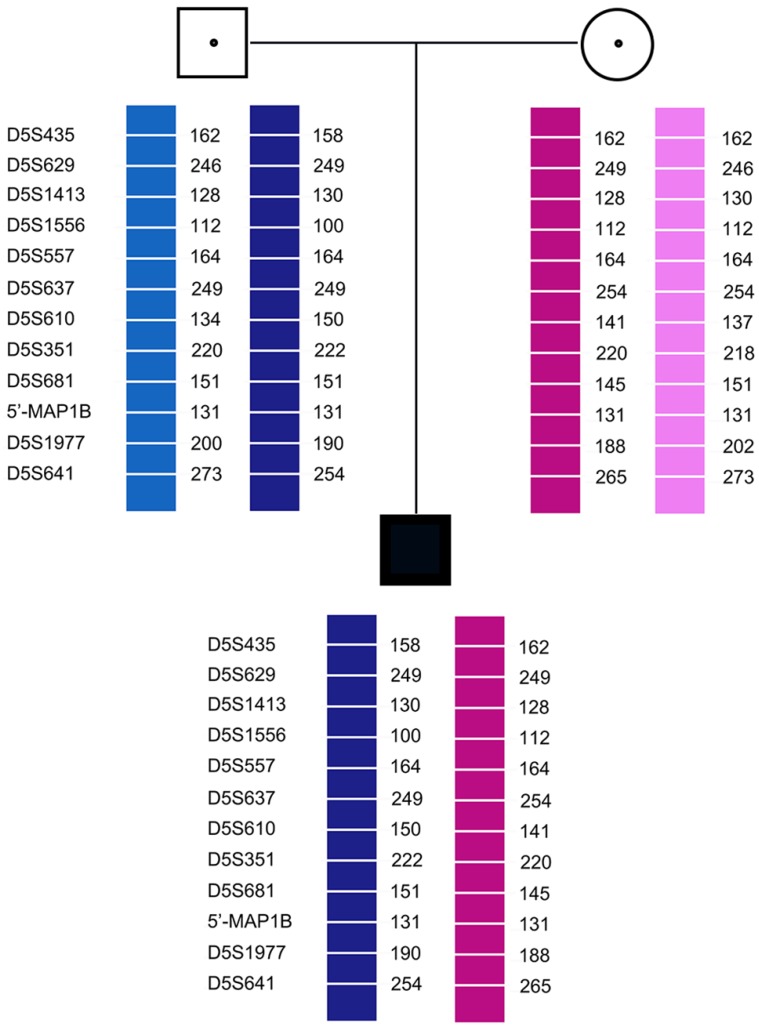
Family analysis.

Pedigree analysis for the twelve families revealed that all carriers were heterozygous for 6-11 polymorphic loci. Genotyping of each pedigree provided 4-9 informative or semi-informative STR markers per couple. The family analyses for the twelve families were shown in Figure 1-3.

### 3.2 Amplification efficiency with MDA

MDA amplification was successful in 93.62% (132/141, range 81.82-100%). Moreover, MDA was successful on 94.57% (87/92) of the single blastomeres and on 91.84% (45/49) of the TE cells.

### 3.3 PCR efficiency and allele drop-out (ADO)

The PCR efficiency were 98.4% (561/570) of the single blastomeres and 100% (182/182) of the TE cells, respectively and the average allele drop-out (ADO) rates were 15.3% (60/392) and 9.8% (11/112), respectively. The results for SMN1 exon 7 were all matched with haplotype analysis, which allowed an accurate diagnosis of 93.61% (132/141) embryos. Two embryos had successful MDA amplification, but because of the large number of ADO in the STR locus, diagnosis could not be performed. And haplotype analysis can be used to identify which embryos were carriers and which embryos were normal.

### 3.4 Clinical PGT-M cycles

Twelve couples were treated from July 2010 to March 2016. A total of 19 ovum pick-ups (16 PGT-M cycles) were performed. Each family had undertaken one to three ovum pick-up cycles. A total of 270 cumulus oocyte complexes were retrieved, and 226 metaphase II oocytes were injected, of which 194 showed normal fertilization.

A total of 141 embryos were tested: 90 embryos were biopsied at the cleavage stage and 51 embryos were biopsied at the blastocyst stage. MDA was successful on 94.44% (85/90) of the single blastomeres and on 92.16% (47/51) of the TE cells. The PCR efficiency were 98.4% (561/570) and 100% (182/182), respectively. The average allele drop-out (ADO) rates were 13.3% (60/392) and 9.8% (11/112), respectively. The results for SMN1 exon 7 were all matched by haplotype analysis, which allowed an accurate diagnosis of 93.62% (132/141) embryos. Five embryos could not be distinguished as carriers or normal due to ADO, but the haplotype analysis showed that at least two STR markers had the normal allele; this was also confirmed by the results for SMN1 exon 7. Therefore, these embryos were suitable for transfer. The number of normal, carrier, and affected embryos were 19, 55, and 53, respectively. Twelve families had unaffected embryos for transfer and a total of 38 embryos were transferred in 20 embryo transfer cycles. Eight transfers were successful, resulting in a clinical pregnancy rate of 40% (8/20) and an implantation rate of 28.95% (11/38). Finally, 11 healthy babies were born. Among them, 5 SMA carriers were singleton live births and and 3 SMA carriers had twin births.

In case 3, recombination of D5S1977 and D5S641 was found in both E1 and E2. In all cases, none of the blank controls showed amplification signals, indicating an absence of contamination.

Genetic testing of the 11 newborns confirmed that none of the babies had an exon 7 homozygous deletion; hence, they were expected to be free of the disease, which matched with the PGT-M results. All 11 infants were developing normally after 2 years of follow-up.

## 4. Discussion

MDA, a non-PCR-based isothermal WGA method, can yield 10^5^ to 10^6^-fold amplifications of DNA from a single cell 24. MDA has a reportedly higher efficiency for more complete and unbiased coverage of the genome when compared to PCR-based WGA methods 24,25. These advantages of MDA provide great potential for expanding PGT-M in the diagnosis of single-gene disorders. For example, Spits et al. 14 reported that a high percentage of the DNA obtained after single-cell MDA was indeed nonspecific; therefore, the yields measured with a spectrophotometer or PicoGreen labeling do not reflect the real efficiency of the technique. Therefore, we considered the PCR efficiency and ADO rates as the criteria for evaluating MDA on single cells rather than yield measurement.

However, MDA has its own drawbacks. For example, the stochastic effects of MDA from single cells are worsened by the nonspecific synthesis and uneven representation of the template due to amplification bias. Another weakness of MDA is that the amplification bias between parental alleles of heterozygous loci may not result from the fidelity of the φ29 DNA polymerase for nucleotide incorporation but instead may arise from the DNA damage and stochastic effects of amplification of a single cell.

The interpretation difficulties caused by ADO events could also lead to misdiagnosis; however, the cause of ADO remains elusive and hard to eliminate. The previously reported levels of ADO in subsequent PCR assays was 25.8% (range 0.0-60.0%), 27.0%, and 12.0% for single lymphocytes and 27.4% for single blastomeres 14,24,28,29. Therefore, a series of informative markers flanking or within the causal gene is required for haplotype analysis to minimize errors due to ADO. When the selected markers were not closely linked to the causal gene, this increased the possibility of rearrangement that led to misdiagnosis or discarding of the available embryos. Simultaneous detection of multiple loci was also labor intensive, time consuming, and costly. In our preliminary study, MDA was successfully performed in 97.2% of a total of 143 single cells, the PCR efficiency was 97.1% (1369/1329) and the allele drop-off (ADO) rate was 9.3% (106/1144), respectively. In clinical applications, the average allele drop-out (ADO) rates were 13.3% (60/392) and 9.8% (11/112), respectively. which was much lower than previously reported rates obtained with MDA and a large number of single cells.

The lower ADO rate in our study may reflect the more careful handling of samples. First, diluted MDA products degrade rapidly with time; therefore, they should be used within 72 hours. Second, during the MDA process, we carefully added different reagents along the wall of the tubes to avoid the formation of bubbles and the use of centrifuges. The mixing of MDA reagents should be completed by gentle tapping rather than vortexing to avoid fragmentation of the MDA products from single cells, as this can increase the ADO rate. Third, all the loci were examined using singleplex fluorescent PCR, which has a lower ADO rate when compared to multiplex PCR. The low ADO rate and direct detection of mutations provided us with a reliable diagnosis of single cells with a small number of highly polymorphic and closely linked loci.

MDA can provide sufficient amounts of DNA for independent PCR assays in PGT-M; thus, it could serve as an alternative approach for assessment of multiple loci or microarray-based aneuploidy screening from single cells. MDA can also provide sufficient leftover DNA for further investigations to confirm which embryo has been implanted and for comparison to the postnatal outcome 30.

Many new methods currently available for PGT-M of SMA, such as karyotyping with single nucleotide polymorphism (SNP) arrays and next generation sequencing (NGS) based on SNP markers, have now been applied to improve the accuracy of PGT-M. However, these methods are expensive, with testing costs amounting to about 4,000 US dollars more than the traditional STR method. In China, PGT-M technology is a self-paying project, which imposes a heavy burden on families with economic difficulties. Moreover, it also requires the PGT-M center to incorporate advanced and expensive equipment, which complicates the spread of new technologies to every PGT-M center. In this study, 11 STR loci were included, which provided sufficient sites for haplotype analysis in most families. As this technology matures, the number of STR loci required for inclusion in the same family during the subsequent PGT-M cycle will be reduced. This will ensure the efficiency of diagnosis while also reducing the workload. For example, the nine STR loci of case 1 were detected during the first PGT-M cycle. We included fully informative and semi-informative markers in the second PGT-M cycle and detected 3 fully informative markers, and all embryos received diagnosis results.

## Conclusion

This study presents a reliable PGT-M method that can be applied to carriers with various mutations of the SMN1 gene for accurate results. It reliably distinguishes carrier embryos from non-carrier embryos and it can be applied to most couples (with deletions or subtle mutations), as segregation of the mutant allele can be followed. Careful performance of the MDA procedure can lower the ADO rates, thereby enabling the use of a small number of highly polymorphic and closely linked loci.

## Figures and Tables

**Table 1:** Patients basic data and embryo biopsy records

Patients NO.	PGD Cycle	Maternal age	Reproductive history	Biopsy time	Embryos biopsy	Successfully expanded by MDA	Normal embryos	Carrier embryos	Abnormal embryos	Unable to judge
NO.1	1	33	G2P2A0	Day3	12	11	1	7	3	
	2	35	G3P3A0	Day5 or Day6	8	8	1	5	2	
NO.2	1	29	G3P2A1	Day3	11	9	1	4	4	
	2	29	G3P2A1	Day3	10	10	2	4	3	1 embryo is unclear whether it is normal or carries
	3	30	G3P2A1	Day3	9	8	3	2	3	
NO.3	1	35	G3P1A2	Day3	3	3	0	1	2	
NO.4	1	34	G2P2A0	Day3	3	3	0	2	1	
NO.5	1	31	G1P1A0	Day3	10	10	1	3	6	
	2	31		Day3	9	9	0	1	4	4 embryos are unclear whether it is normal or carries
NO.6	1	27	G2P2A0	Day3	10	9	2	2	5	
NO.7	1	34	G3P1A2	Day5 or Day6	16	13	3	6	4	
NO.8	1	36	G1P1A0	Day5 or Day6	5	5	1	2	2	
NO.9	1	32	G4P2A1	Day5 or Day6	6	6	0	4	2	
NO.10	1	30	G1P1A0	Day3	15	15	2	5	8	
NO.11	1	43	G3P1A1	Day5 or Day6	4	4	0	3	1	
NO.12	1	36	G2P1A1	Day5 or Day6	10	9	2	4	3	

**Table 2:** Primer sequence

	Forward primers (5'-3')	Reverse primer (5'-3')
D5S435	FAM-TGGTCCTAAGATAGGGTTGAT	CAAGAGCACAGTTTGGAGTGAG
D5S629	Hex-ACTCGGGAGGCTGAGA	CCGGTTTGTTCCTGTGA
D5S1413	TAMRA-GCTACAGGCCAGATGAGGGAAATAG	AAAATAGGCTTGTGAAACCAACGC
D5S1556	Hex-ATTTACTTTTCCAAGGGGGAGG	CATGTTGCTTAGGCCTCGTCT
D5S610	FAM-GGCAGTGTCCTAAAATCTTTTG	CCTAAACTGAACTTTCAAAGCTG
D5S637	TAMRA-TGAATCTCAGGGAGTTGTGAA	CTGCATTTAATACTGCAATGAA
D5S557	FAM-AAGTGAAACACAGAGGTTGAC	GGTGAATGTTTGATGACCCTA
D5S351	Hex-AAGACCAGTCTATGGCAACAC	GTGAGACCGAAAATGCTGATG
D5S681	FAM-ATCTCTGAGGCTGCACAT	GTCTTTGATGAGATACCG
5'-MAP1B	FAM-TCCTTCTTCCAAAACCAGGGTGAAGCCTC	AAATTTCTAGGATGCTTGCGGGATCTCTTC
D5S1977	TAMRA-TGACAAAGCAAGGCTCTC	GATGTTCATCAGACTCAGAACC
D5S641	FAM-AGGGACAGTCCACTTCCAGT	AGTTGTGTATTGGAGAATGTTATCA
SMN1exon 7	FAM-AGACTATCAACTTAATTTCTGATCA	TCCTTCTTTTTGATTTTGTCTG

**Table 3:** Pregnancy outcomes

Patients NO.	Ovum retrieval cycle	PGT-M cycle	Embryo transfer cycle	NO. of transplanted embryos	Pregnancy	NO. of fetal hearts	NO. of Live birth
1	3	2	1	3	YES	1	1
			2	3	NO	0	0
			3	2	YES	2	1
2	3	3	1	3	NO	0	0
			2	2	NO	0	0
			3	2	YES	2	2
3	1	1	1	1	NO	0	0
4	1	1	1	1	NO	0	0
5	2	2	1	2	NO	0	0
			2	1	YES	1	1
6	2	1	1	2	NO	0	0
7	1	1	1	2	YES	2	2
8	2	1	0	0	NO	0	0
9	2	1	1	2	NO	0	0
			2	2	YES	1	1
10	1	1	1	2	NO	0	0
			2	2	YES	1	1
11	2	1	1	1	NO	0	0
			2	1	NO	0	0
			3	1	NO	0	0
12	1	1	1	1	YES	1	1
